# Predictors of Mental Health Outcomes in Road Traffic Accident Survivors

**DOI:** 10.3390/jcm9020309

**Published:** 2020-01-22

**Authors:** Jelena Kovacevic, Maja Miskulin, Dunja Degmecic, Aleksandar Vcev, Dinko Leovic, Vladimir Sisljagic, Ivana Simic, Hrvoje Palenkic, Ivan Vcev, Ivan Miskulin

**Affiliations:** 1 Institute of emergency medicine of the Vukovar-Srijem County, 32 100 Vinkovci, Croatia; dr.kovacevic.jelena@gmail.com; 2 Faculty of Medicine Osijek, Josip Juraj Strossmayer University of Osijek, 31 000 Osijek, Croatia; maja.miskulin@mefos.hr (M.M.); ddegmecic@gmail.com (D.D.); aleksandar.vcev@fdmz.hr (A.V.); dinko.leovic@gmail.com (D.L.); sisljagic.vladimir@kbo.hr (V.S.); ivana.simic.osijek@gmail.com (I.S.); hrpal@net.hr (H.P.); 3 Department of Psychiatry, University Hospital Osijek, 31000 Osijek, Croatia; 4 Faculty of Dental Medicine and Health Osijek, Josip Juraj Strossmayer University of Osijek, 31 000 Osijek, Croatia; 5 Department of Internal Medicine, University Hospital Osijek, 31000 Osijek, Croatia; 6 Department of Surgery, University Hospital Osijek, 31000 Osijek, Croatia; 7 Institute of Public Health for the Osijek-Baranja County, 31 000 Osijek, Croatia; 8 Department of Surgery, General Hospital Slavonski Brod, 35 000 Slavonski Brod, Croatia; 9 Department of Humanities, University of Zadar, 23 000 Zadar, Croatia; ivan_vcev@student.unizd.hr

**Keywords:** road traffic accident, posttraumatic stress disorder, depression, anxiety, injury severity

## Abstract

Mental health outcomes of road traffic accidents (RTAs) are always investigated in assessments of those involved. The aim of this study was to investigate the psychological consequences and associated factors in all RTA survivors, irrelevant of their injury status. A cohort of 155 people was assessed one month after experiencing a RTA using self-reported measures for posttraumatic stress disorder (PTSD), depression, and anxiety. Associations between mental health outcomes and sociodemographic factors, pre-RTA health status, injury-related factors, and RTA details were analyzed. RTA survivors reported substantial rates of PTSD (32.3%) and depression (17.4%) symptoms, and low rates of anxiety (5.8%). Symptoms of depression were associated with below-average self-perceived economic status, irreligiousness, medication use, psychiatric medication use, and injury-related factors. PTSD symptoms were associated with female gender, below-average self-perceived economic status, previous psychiatric illness, medication use, psychiatric medication use, not being at fault in the relevant RTA, claiming compensation, and injury-related factors. Anxiety symptoms were associated with previous chronic or psychiatric illness, previous permanent pain, psychiatric medication use, and self-perceived threat to life, but not with sustaining injury. Along with the evaluation and treatment of RTA injuries, health care providers should evaluate the pre-RTA health status of all RTA victims. Psychological support to those at risk may prevent psychological disorders after RTAs.

## 1. Introduction

The World Health Organization (WHO) estimated that up to 50 million people worldwide sustain nonfatal injuries in road traffic accidents (RTAs) every year [1]. RTAs significantly contribute to the global burden of disease, being the fifth leading cause of disability-adjusted life years (DALYs) in the world [2]. Globally, an estimated 3% of GDP is lost to road traffic deaths and injuries [3]. In 2018 in Croatia, 317 people died and 13989 were injured in RTAs [4]. Around 80% of RTA victims in Croatia sustain minor injuries and 5% of the heavily injured become permanently disabled [5]. 

Nonfatal consequences of RTAs have numerous dimensions, e.g., functional impairment, cognitive dysfunction, psychological suffering, and the loss of quality of life of the victims and their families [6]. A significant number of RTA survivors will develop psychological disorders, the most common being posttraumatic stress disorder (PTSD), depression, and anxiety disorders [7,8,9,10,11,12,13,14,15,16,17,18,19,20]. Psychological consequences are often overlooked due to a primary focus on physical injuries, although research has shown that psychological consequences following RTAs can be long-term, and that psychological and physical outcomes interact and influence one another [21]. Studies have shown that RTA survivors do not fully recover, even years after the RTA [18,22].

RTAs are the leading cause of PTSD in the general population [23]. PTSD prevalence after a RTA ranges from 6% to 45%, depending on the time frame and sample size, as well as the socioeconomic and cultural factors of the country where study was conducted [21]. The WHO World Mental Health Survey Initiative determined an overall PTSD rate of 2.5% after any RTA that was perceived to be life-threatening in a community-based epidemiological survey conducted in 13 countries, but concluded that even a relatively low prevalence of PTSD after RTAs represents a significant global public health problems, given the enormous number of RTAs that occur worldwide [24]. A recent meta-analysis found pooled prevalence of PTSD among RTA survivors to be 22.25%, with great disparity across studies in relation to the instruments used to assess PTSD, country, race, gender, and education level [17]. Consistent predictors of PTSD are rumination about the trauma, perceived threat to life, lack of social support, severe acute stress disorder symptoms, persistent physical problems, previous emotional problems, previous anxiety disorders, and involvement in litigation/compensation [21]. Results regarding the association between the RTA injury level and PTSD are contradictory and demand more research [6,21]. 

Research regarding other psychological disorders after a RTA, such as depression and anxiety, are not as numerous as for PTSD. The obtained prevalence for depressive disorder after a RTA ranges from 7.8% to 63% [8,9,11,12,13,15,16,20,25,26], and for anxiety disorder from 19.4% to 60% [9,25]. There are no meta-analyses for depression and anxiety after RTAs, nor for the predictors of these disorders after a RTA.

Research has shown that sociodemographic and health-related factors prior to a RTA are associated with unfavorable functional outcomes [27]. Understanding the prevalence and nature of psychological disorders after RTAs is essential for introducing the most efficient and timely intervention. Early detection of susceptible individuals would enable interventions that would facilitate optimal recovery after a RTA [21]. Therefore, investigating the long-term consequences of a RTA is essential for designing institutional responses to the recovery process of RTA survivors [22].

Very few studies have investigated health-related outcomes of RTA survivors in Europe [6,8,16,20], and to the best of our knowledge, none have been conducted in Croatia investigating the physical or psychological consequences for RTA survivors, including psychological disorders such as PTSD, depressive disorder, and anxiety disorder. Therefore, little is known about the factors that influence the recovery of RTA survivors in this population. 

A recent meta-analysis concluded that psychological distress following a RTA is substantial, but was unable to determine whether psychological distress is elevated by the injury and/or the trauma of the accident [18], since no studies have been conducted on RTA survivors who had not sustained injuries. However, it is likely that psychological distress is elevated when people experience a traumatic accident, even when no injuries were sustained [18]. 

Therefore, the aim of this study was to investigate the psychological consequences of all RTA survivors in Croatia, irrelevant of their injury status, and to explore the factors contributing to unfavorable mental health outcomes. 

## 2. Materials and Methods 

This was a prospective study of a cohort of 155 people who had experienced a RTA in Croatia. The assessment was conducted 1 month after the RTA. RTA survivors were recruited from the database of the Institute of emergency medicine of the Vukovar-Srijem County in Croatia in the period from October 2016 to September 2017. Both RTA survivors sustaining injuries and those without injuries were contacted. Data were collected in person by a medical doctor. All participants signed an informed consent. The research was approved by the Ethics Committee of the Faculty of Medicine Osijek, Croatia (Ethical Approval Code: 2158-61-07-17-211). 

Eligibility for participation in the study was based on the following inclusion criteria: recent RTA experience, being 18 years of age or more, and consent for participation in the study. Exclusion criteria were as follows: major head trauma with subsequent cognitive impairment, previous cognitive impairment affecting ability to offer consent and understand questions asked, and aged under 18 years. In addition to self-reported information, all participants provided their medical records regarding RTAs. 

**Figure 1:**
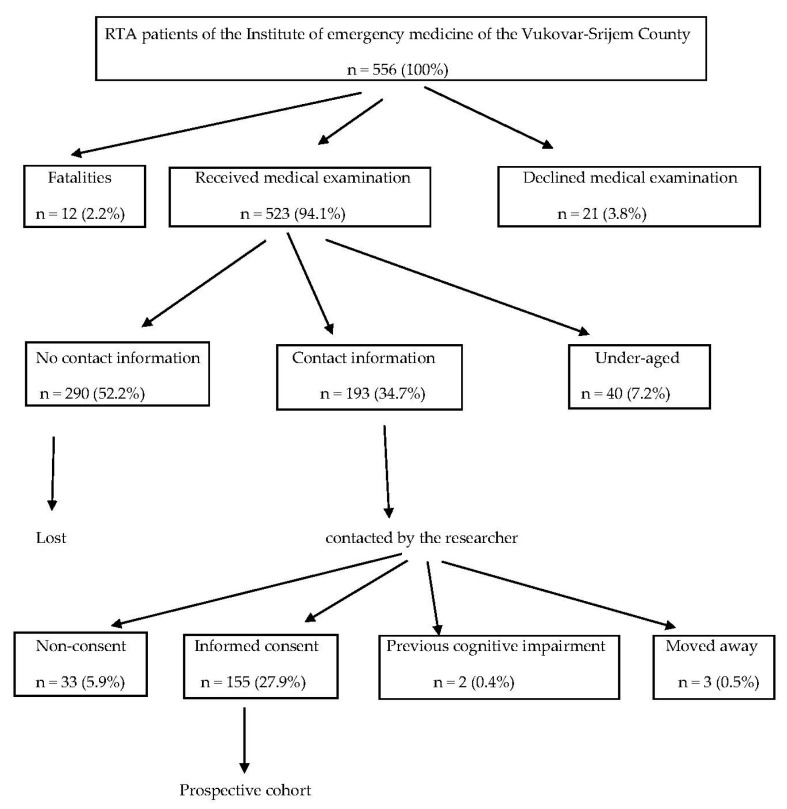
Cohort recruitment diagram.

During the research period, 556 people were registered at the Institute of emergency medicine of the Vukovar-Srijem County as RTA victims. Of those, 12 (2.2%) were fatalities, 21 (3.8%) declined medical care, and 40 (7.2%) did not meet the inclusion criteria since they were under-aged. For 290 (52.2%) patients, no contact information was available. Therefore, 193 (34.7%) patients were contacted by telephone. From among them, 33 (5.9%) declined to participate, 3 (0.5%) had since changed address, and 2 (0.4%) did not meet the inclusion criteria due to previous cognitive impairment. None of the contacted RTA survivors met the exclusion criterion of major head trauma with subsequent cognitive impairment and inability to give consent for participation in the study. Finally, 155 (27.9%) people gave informed consent to participate in the study. Details of the participant recruitment process is presented in Figure 1.

Sociodemographic assessment included age, sex, residency (urban/rural), education level (primary/secondary/university), employment status (employed/unemployed/retired), marital status (single/having a partner), self-perceived economic status (below average/average/above average), and religiousness (yes/no). Lifestyle and health-related factors included smoking, alcohol and psychoactive substance use, medication use, previous RTA experience, previous traumatic experience, previous PTSD, previous chronic illness, previous psychiatric illness, pre-RTA pain. These were dichotomized as yes/no answers. 

RTA details included the road user type, total number of motor vehicles engaged, total number of victims and/or deaths, fault in the RTA, compensation status, amnesia from the RTA, unconsciousness in the RTA, injuries sustained, hospitalization duration, surgical treatment after the RTA, rehabilitation after the RTA, self-perceived threat to life in the RTA, pain location, and pain frequency after the RTA. 

The severity of injuries was based on the Abbreviated Injury Scale (AIS) that categorizes injuries as minor—1, moderate—2, serious—3, severe—4, critical—5 and fatal—6 [28]. To assign a final score, the New Injury Severity Scale (NISS) was used, computed as the sum of squares of the three most severe AIS scores, irrespective of body region. The obtained values were categorized as minor (NISS < 4), moderate (NISS 4–8), serious (NISS 9–15), severe (NISS 16–24), and critical (25–75) [29].

PTSD symptoms were assessed using the PTSD Checklist for civilians (PCL-C) [30]. PCL-C is a 17-item self-reporting instrument reflecting the Diagnostic and Statistical Manual of Mental Disorders Fourth Edition (DSM-IV) symptoms of PTSD. Responses range from 1 to 5, and the total score is computed by summing all items. The cut-off point used depends on the goal of assessment and the prevalence of PTSD in the target setting. A cut-off point of 30 is suggested for general population samples [31]. 

Anxiety disorder was assessed using the Beck Anxiety Inventory (BAI) [32]. BAI is a 21-item, self-report measure of anxiety that assesses anxiety symptoms over the past month using a four-point Likert scale. Total score may range from 0 to 63. The classification of anxiety scores is as follows: 0–21 low anxiety, 22–35 moderate anxiety, ≥36 potentially concerning levels of anxiety. The cut-off point for the presence of anxiety symptoms was 22.

Depressive disorder was assessed using the Beck Depression Inventory—I (BDI—I) [33]. BDI—I is a validated 21-item, self-reporting scale that assesses depression symptoms over the past month using a four-point Likert scale. Total score may range from 0 to 63. The classification of depression scores is as follows: 0–10 normal, 11–16 mild mood disturbance, 17–20 borderline clinical depression, 21–30 moderate depression, 31–40 severe depression, and >40 extreme depression. The cut-off point for the presence of depression symptoms was 11.

Following confirmation of the normality of the data distribution by the Kolmogorov–Smirnov test, all data were processed by the methods of descriptive statistics. The numerical variables were described as median and interquartile range. The categorical variables were described in absolute and relative frequencies. The *χ*^2^-test and Fisher exact test were used for the comparison of categorical variables between the groups. The level of statistical significance was set at *p* < 0.05. Statistical analysis was done using the statistical package Statistica for Windows 2010 (version 10.0, StatSoft Inc., Tulsa, OK, USA).

## 3. Results

### 3.1. Sociodemographic Characteristics of the Participants before the RTA

The median age of the participants was 42 years (interquartile range 27.5–56.0), and 54.8% were male; 41.9% of the participants had rural and 58.1% had urban residences; 67.1% of the participants had completed secondary education, 14.8% had graduated from university, and 18.1% had completed or incomplete primary education; 58.1% were employed, 27.7% were unemployed, and 14.2% were retired. Marital status was 38.1% of the participants self-reporting as single, while 61.9% reported having a partner. Self-perceived economic status was as follows: 59.3% were average, 21.3% were above average, and 19.4% were below average. Smokers made up 37.4% and alcohol users 51.0%. Only 0.6% reported using psychoactive substances. Concerning religion, 89.7% reported that they practiced a faith; 51.6% reported using medications, 3.2% reported using psychiatric medications, 40.6% reported using nonpsychiatric medications, and 7.7% reported using both types of medications; 41.3% of participants had previous RTA experience and 52.9% had previous traumatic experience. Previous PTSD was reported by 3.2%, previous chronic illness by 41.9%, and previous psychiatric illness by 11.6% of the participants. Previous permanent pain was reported by 9.7% of the participants (Table 1). 

### 3.2. RTA-Related Characteristics of the Participants

Motor vehicle drivers accounted for 58.7% of the participants, 32.9% were codrivers or passengers, and 8.4% were cyclists or pedestrians; 47.1% reported one motor vehicle being involved in the RTA, and 52.3% reported two or more motor vehicles being involved. As for the total number of injured people in the RTA, 43.3% reported one injured person, 46.5% reported two or more injured people, and 10.3% reported no one injured. Only 1.3% reported fatalities in the RTA. Fault for causing the RTA was reported by 35.5%, and no fault was reported 61.9%. Unknown fault was reported by 2.6% of the participants where fault in the RTA was not yet established in a court of law. Claiming compensation was reported by 42.6% of the participants and 8.4% reported receiving compensation (Table 2).

### 3.3. RTA Injury Characteristics

Multiple injuries were reported by 61.9% of the participants, while 26.5% reported only one injury; 56.9% of participants reported injuries in multiple body regions. The most common primary site of injury was the head (31.1%), followed by neck (18.7%), legs (13.5%), thorax (10.3%), hands (9.0%), and abdomen (5.8%). No injuries were reported 11.6% of the participants. Injury severity of the participants was as follows: 52.9% sustained mild injuries, 16.2% sustained moderate injuries, 14.8% sustained serious injuries, 3.2% sustained severe injuries, 1.3% sustained critical injuries, and 11.6% sustained no injuries. Serious, severe, and critical injuries were analyzed as one category. Self-perceived threat to life was reported by 43.2% of RTA survivors; 45.8% reported pain after the RTA in several body parts, 32.3% reported pain in one specific body part, and 21.9% reported no pain after the RTA. Pain frequency was reported as permanent in 27.1%, occasional in 31.6%, and circumstantial in 19.4% of the participants. Loss of consciousness in the RTA was reported 16.8%, and post RTA amnesia was reported 15.5%. Hospitalization due to RTA was reported by 32.3%. Surgical treatment was reported by 9.7% and rehabilitation procedures were reported by 23.2% of the participants (Table 3).

Injuries were more frequent (*p* = 0.006) and severe (*p* = 0.010) among those who perceived their economic status as being below average. Self-perceived threat to life was associated with sustaining injury in the RTA (*p* = 0.001) and with injury severity (*p* = 0.002). Pain after the RTA was associated with injury affliction (*p* < 0.001) and severity (*p* < 0.001). Rehabilitation procedures were associated with sustaining injury (*p* = 0.014) and injury severity (*p* = 0.010). Other factors not found to be associated with injury affliction or severity are presented in Table 4.

### 3.4. Psychological Consequences of the RTA

Depression symptoms were reported by 17.4% (95% CI: (11.4–23.4)), anxiety symptoms by 5.8% (95% CI: (2.1–9.5)), and PTSD symptoms by 32.3% (95% CI: (24.9–39.7)) of the participants (Table 3). The cooccurrence of PTSD and depression symptoms was found in 12.9% (95% CI: (7.6–18.2)) of participants, PTSD and anxiety symptoms in 28.4% (95% CI: (21.3–35.5)), while anxiety and depression symptoms were found in 14.2% (95% CI: (8.7–19.7)) of the participants. A comorbidity of PTSD, depression, and anxiety symptoms was found in 11.0% (95% CI: (6.1–15.9)).

Depression symptoms were associated with self-perceived economic status as being below average (*p* = 0.001), irreligiousness (*p* < 0.001), medication use (*p* = 0.001), especially psychiatric medication use (*p* = 0.002), injury severity (*p* < 0.001), unconsciousness in the RTA (*p* = 0.020), post-RTA amnesia (*p* = 0.015), hospitalization after RTA (*p* = 0.006), surgical treatment after RTA (*p* = 0.001), and duration of hospitalization (*p* = 0.009). Other factors that were not associated with depression symptoms are presented in Table 5. 

Anxiety symptoms were associated with previous chronic illness (*p* = 0.035), previous psychiatric illness (*p* = 0.011), psychiatric medication use (*p* = 0.010), previous permanent pain experience (*p* < 0.001), and self-perceived threat to life (*p* = 0.040). Other factors not found to be associated with anxiety symptoms are presented in Table 5.

PTSD symptoms were associated with female gender (*p* = 0.038), below-average self-perceived economic status (*p* = 0.025), medication use (*p* = 0.002), especially psychiatric medication use (*p* < 0.001), previous psychiatric illness (*p* = 0.008), RTA injury affliction (*p* = 0.013), injury severity (*p* = 0.004), self-perceived threat to life (*p* = 0.002), post-RTA pain (*p* = 0.006), hospitalization duration (*p* = 0.011), not at fault in the RTA (*p* = 0.044), and claiming compensation (*p* < 0.001). Other factors not found to be associated with PTSD symptoms are presented in Table 5. 

## 4. Discussion

This study explored the psychological consequences of experiencing a RTA among survivors sustaining injuries and those without injuries. The cohort was characterized by high levels of PTSD and depression symptoms, and low levels of anxiety. The obtained prevalence results were in the expected range, and similar to those of other studies of PTSD [8,9,11,14,15,16,17,20,21] and depression [11,12,14,26] among RTA survivors. Other studies obtained higher prevalence rates of depression and/or anxiety [8,9,13,15,16,20,25,34], but prevalence rates must be viewed relative to the time-point, sample, and instruments used [17]. 

Comorbidity of investigated disorders was also found in other studies [9,11,14,15,16]. RTA victims with comorbid psychological disorders should be given early special attention, since comorbidity was found to be predictive of long-term mental health-related outcomes [9]. 

Injury affliction in the RTA and injury severity were associated with below-average self-perceived economic status. Since 91.6% of the participants were vehicle occupants, we can presume that people of lower economic status drive less safe vehicles with fewer safety features, and therefore, sustain injuries more often and more severely. In the last decade, crash data confirmed that a 50% reduction in the risk of serious injury has been achieved in new car models [35]. However, the average age of registered vehicles in Croatia is 13.68 years [36].

Sociodemographic factors explored in this study showed that the most important pre-RTA characteristic of the participants associated with the symptoms of psychological disorders after a RTA is the health status beforehand, i.e., previous chronic illness, previous psychiatric illness, previous permanent pain, and previous medication use. Psychiatric medication use before the RTA was associated with symptoms of all three psychological disorders investigated. The significance of this factor should be recognized and further explored, since it is possible that some people are reluctant to report previous psychiatric illness, but more readily give information about medications they use for their health conditions. Health status before the RTA was also determined as an important factor for developing psychological disorders in other studies [7,9,21,34]. Therefore, study results indicate that people with previously deteriorated health are at greater risk of experiencing negative psychological consequences from a RTA, irrelevant of injury related factors. 

Other significant sociodemographic factors determined in this study were female gender for PTSD symptoms, below-average self-perceived economic status for PTSD and depression symptoms, and irreligiousness for depression symptoms. Females were found to be more susceptible to psychological disorders after RTA, namely PTSD, in other studies [7,11,14,16,17,37,38]. The observed difference is explained by different coping strategies and trauma interpretations in women [17].

There has been disagreement in the literature regarding whether or not injury severity predicts PTSD due to contradictory research data [6,21]. This study showed that injury severity was associated with PTSD symptoms, as did many other studies [6,7,16,19,20]. Furthermore, significant difference in the presence of PTSD symptoms was found among injured and uninjured RTA survivors, which corroborates the importance of sustaining injury in the development of PTSD symptoms. Data regarding the impact of injury and injury severity on other psychological disorders are scarce and lacking meta-analyses. This study showed the association of injury severity and depression symptoms, but not anxiety symptoms. Few other studies also associated injury severity with depression [16,20], but others found no association between injury severity and depression and/or anxiety [8,26,39]. Contradicting results might arise from the fact that most research is restricted to certain levels of injuries [21], and that different researchers use different injury severity scales. Moreover, none of the previous research involved uninjured RTA survivors that present a valuable group of RTA victims who had experienced a traumatic event without being physically injured. 

Factors indirectly associated with the injury were also associated with the symptoms of psychological disorders after a RTA. This study, like others, found pain after the RTA to be associated with injury severity [6] and PTSD symptoms [7,11,40]. Hospitalization, surgical treatment, unconsciousness in the RTA, and post-RTA amnesia were associated with depression symptoms, and hospitalization duration was associated with both PTSD and depression symptoms. Others also found injury-related factors, such as hospitalization [16] and post-traumatic amnesia [7], to be associated with psychological consequences after a RTA.

Self-perceived threat to life was associated with injury affliction and injury severity, and with symptoms of PTSD and anxiety, but not with depression symptoms. Similarly, others also found this factor to be associated with PTSD [13,19] and not with depression [13]. Some found self-perceived threat to life to be associated with higher odds of experiencing both anxiety and depression [41]. 

RTA details found significant for psychological outcomes were not being at fault in the RTA and compensation claims that were associated with PTSD symptoms. Others also found that not-at-fault RTA survivors showed more emotional and mental disturbance than those at fault [7,25,38]. Involvement in the compensation process after a RTA was recognized as a predictor of PTSD in other studies [21,42], and was associated with higher anxiety in RTA survivors [39]. However, the influence of compensation on RTA outcomes is controversial, because it is not clear whether it is the compensation itself that is associated with limited recovery or factors associated with people who claim compensation [39]. The compensation process may serve as a constant reminder of the RTA and traumatic details through the necessity of dealing with unsupportive or stressful insurers, for example [21]. Strategies for minimizing stress during the compensation process may help RTA survivors [18]. Unlike this study, few studies found fatalities in the RTA to be predictor of PTSD [21,24]; this disparity might be due to the fact that only 1.3% of the participants in this study experienced fatalities in the RTA. 

Previous research has suggested a relationship between PTSD and depression [11,14,16]. This study found that factors associated with PTSD and depression symptoms are similar and mostly injury-related. This should be a guideline for the further research and development of appropriate screening tools and interventions for those RTA victims with such attributes. Possible screening setting might be health-care based, where injured RTA survivors would be available for screening, e.g., hospital trauma units and rehabilitation centers. Available psychological counselling at trauma centers and hospitals may facilitate the recovery of RTA survivors [43], especially since it was found that RTA survivors with PTSD were more likely to meet diagnostic criteria for any other mental disorder in the long-term [9].

Anxiety symptoms showed significant association with previous health status, i.e., previous chronic illness, previous psychiatric illness, previous permanent pain, and psychiatric medication used before a RTA, but not with injury-related factors. Furthermore, there were 11.1% of participants with depression symptoms, 5.6% with PTSD symptoms, and 5.6% with anxiety symptoms among uninjured RTA survivors. These results implicate the need for further studies involving uninjured RTA survivors as a neglected population of RTA victims. Among them might be individuals with previous physical and mental health conditions who might be at risk of developing psychological disorders after a traumatic experience such as a RTA.

This study showed possible directions of future research involving RTA victims and indicated possible risk factors for RTA survivors who are at significant risk for developing symptoms of psychological disorders after experiencing a RTA. Such vulnerable RTA victims should be the target of possible interventions developed to prevent negative psychological consequences after a RTA.

### Strengths and limitations

Sociodemographic data and screening tools were self-reported, with the attempt to mitigate this by a medical doctor collecting the data in person. Furthermore, participants were asked about preexisting physical and mental health problems, but specific diagnostic tools or medical records other than those related the RTA were not used.

The recruited cohort comprised only 27.9% of registered RTA victims, not due to a low response rate, but largely due to the lack of contact information of the RTA survivors (52.2%). The response rate was very high, i.e., 80.3% of those who were contacted agreed to participate. This raises the question of obtaining contact information, i.e., obtaining telephone contact details is not part of the routine protocol in the institutions of emergency medicine in Croatia, since the patients are provided with emergency medical care at the scene of the RTA and in the emergency medical vehicle. Thereafter, they are transported to hospitals where they are subsequently provided healthcare. There are also objective factors that make it difficult to obtain contact information in an emergency medical vehicle, e.g., unconsciousness or other medical conditions of the patient, the absence of a family member or friend, etc.

The strength of this study is in the fact that the cohort included RTA victims with all levels of injury severity and uninjured RTA survivors. Also, the participants were recruited from the public health-care system that provides emergency medical care to all RTA victims in Croatia, regardless of their health insurance status, unlike many studies that recruited participants from compensation claim registers [9,13,34,38,41,42] or only hospitalized patients [8,10,12,20,42,43]. Therefore, this cohort may represent the general Croatian population. The major limitation of the study is the relatively small sample size. Future research may benefit from a larger sample size and more resources being allocated to the recruitment of participants, i.e., obtaining more comprehensive contact information.

## 5. Conclusions

A RTA is a traumatic event that can result in physical injuries, but also in psychological consequences depending on the pre-RTA survivor’s characteristics. Understanding the factors that present risk for poor mental health outcomes after the RTA is a key step in planning and organizing the recovery of RTA survivors. Depending on the predictors used, the appropriateness of the timing and screening setting should be considered. Along with the evaluation and treatment of RTA injuries, health care providers should evaluate the pre-RTA health status of all RTA survivors, regardless of their injury status. Psychological support to those at risk may prevent psychological disorders from developing after a RTA.

## Figures and Tables

**Table 1:** Pre-RTA characteristics of the participants.

	N	%
**Gender**		
Male	85	54.8
Female	70	45.2
**Age group (years)**		
Younger (18–41)	77	49.7
Older (≥42)	78	50.3
**Residence**		
Urban	65	41.9
Rural	90	58.1
**Education level**		
Primary	28	18.1
Secondary	104	67.1
University	23	14.8
**Employment**		
Employed	90	58.1
Unemployed	43	27.7
Retired	22	14.2
**Marital status**		
Single	59	38.1
With a partner	96	61.9
**Self-perceived economic status**		
Below average	30	19.4
Average	92	59.3
Above average	33	21.3
**Smoking**		
No	97	62.6
Yes	58	37.4
**Alcohol use**		
No	76	49.0
Yes	79	51.0
**Psychoactive substance use**		
Never	154	99.4
Sometimes	1	0.6
**Religiousness**		
No	16	10.3
Yes	139	89.7
**Medication use**		
No	75	48.4
Yes	80	51.6
**Type of medication used**		
None	75	48.5
Nonpsychiatric	63	40.6
Psychiatric	5	3.2
Both types	12	7.7
**Previous RTA experience**		
No	91	58.7
Yes	64	41.3
**Previous traumatic experience**		
No	73	47.1
Yes	82	52.9
**Previous PTSD**		
No	150	96.8
Yes	5	3.2
**Previous chronic illness**		
No	90	58.1
Yes	65	41.9
**Previous psychiatric illness**		
No	137	88.4
Yes	18	11.6
**Previous permanent pain**		
No	140	90.3
Yes	15	9.7

**Table 2:** RTA-related characteristics of the participants.

	N	%
**Road user type**		
Motor vehicle driver	91	58.7
Codriver/passenger	51	32.9
Cyclist/pedestrian	13	8.4
**Motor vehicles engaged in the RTA**		
None	1	0.6
One	73	47.1
More than one	81	52.3
**Total number of injured people in the RTA**		
None	16	10.3
One	67	43.3
2–3	58	37.4
4–5	14	9
**Fatalities in the RTA**		
No	153	98.7
Yes	2	1.3
**Fault in the RTA**		
No	96	61.9
Yes	55	35.5
Unknown	4	2.6
**Compensation claim after the RTA**		
No	89	57.4
Yes	66	42.6
**Received compensation after the RTA**		
No	142	91.6
Yes	13	8.4

**Table 3:** RTA injury characteristics and psychological consequences.

	N	%
**Number of injuries in the RTA**		
None	18	11.6
One	41	26.5
Multiple	96	61.9
**Location of injuries in the RTA**		
None	18	11.6
One: Head	18	11.6
Face	2	1.3
Neck	7	4.5
Thora	7	4.5
Abdomen	1	0.6
Spine	3	1.9
Hands	3	1.9
Legs	8	5.2
Multiple body parts	88	56.9
**Primary site of injury**		
None	18	11.6
Head	48	31.1
Neck	29	18.7
Thorax	16	10.3
Abdomen	9	5.8
Hands	14	9
Legs	21	13.5
**Injury severity**		
No injury	18	11.6
Mild	82	52.9
Moderate	25	16.2
Serious	23	14.8
Severe	5	3.2
Critical	2	1.3
**Self-perceived threat to life**		
No	88	56.8
Yes	67	43.2
**Unconsciousness in the RTA**		
No	129	83.2
Yes	26	16.8
**Post-RTA amnesia**		
No	131	84.5
Yes	24	15.5
**Hospitalization**		
No	105	67.7
Yes	50	32.3
**Hospitalization duration**		
0 days	105	67.8
1–3 days	21	13.5
4–10 days	16	10.3
Over 10 days	13	8.4
**Surgical treatment**		
No	140	90.3
Yes	15	9.7
**Rehabilitation after RTA**		
No	119	76.8
Yes	36	23.2
**Pain location after RTA**		
No pain	34	21.9
Certain body part	50	32.3
Multiple body parts	71	45.8
**Pain frequency after RTA**		
Never	34	21.9
Circumstantial	30	19.4
Occasional	49	31.6
Permanent	42	27.1
**PTSD symptoms**		
No	105	67.7
Yes	50	32.3
**Depression symptoms**		
Normal mood	128	82.6
Mild mood disturbance	20	12.9
Borderline clinical depression	7	4.5
**Anxiety symptoms**		
Low	146	94.2
Moderate	8	5.2
Concerning	1	0.6

**Table 4:** Factors associated with RTA injuries.

	Injury Affliction (yes/no)	Injury Severity (NISS)
Gender	*p* = 0.623^a^	*p* = 0.788^a^
Age	*p* = 0.453^a^	*p* = 0.245^a^
Residency	*p* = 0.612^a^	*p* = 0.069^a^
Education level	*p* = 0.242^b^	*p* = 0.270^b^
Employment status	*p* = 0.124^b^	*p* = 0.092^b^
Self-perceived economic status	*p* = **0.006^b^**	*p* = **0.010^b^**
Smoking	*p* > 0.999^a^	*p* = 0.937^a^
Alcohol use	*p* = 0.211^a^	*p* = 0.257^a^
Drug use	*p* > 0.999^b^	*p* > 0.999^b^
Religiousness	*p* = 0.219^b^	*p* = **0.047^b^**
Medication use	*p* = 0.318^a^	*p* = 0.247^a^
Type of medication used	*p* = 0.563^b^	*p* = 0.600^b^
Previous RTA experience	*p* = 0.454^a^	*p* = 0.708^a^
Previous traumatic experience	*p* > 0.999^a^	*p* = 0.933^a^
Previous PTSD	*p* > 0.999^b^	*p* = 0.697^b^
Previous chronic illness	*p* = 0.216^a^	*p* = 0.403^a^
Previous psychiatric illness	*p* = 0.697^b^	*p* = 0.622^b^
Previous permanent pain	*p* = 0.687^b^	*p* = 0.930^b^
Self-perceived threat to life	*p* = **0.001^a^**	*p* = **0.002^a^**
Pain after RTA	*p* < **0.001^b^**	*p* < **0.001^b^**
Road user type	*p* = 0.088^b^	*p* = 0.053^b^
Rehabilitation after RTA	*p* = **0.014^b^**	*p* = **0.010^b^**
Compensation claim	*p* = 0.804^a^	*p* = 0.860^a^
Received compensation	*p* = 0.649^b^	*p* = 0.138^b^

^a^ Chi-square test; ^b^ Fisher’s exact test. Bold *p* values are statistically significant.

**Table 5:** Factors associated with psychological consequences after RTA.

	Depression Symptoms	Anxiety Symptoms	PTSD Symptoms
**Sociodemographic**			
Gender	*p* = 0.525^a^	*p* = 0.079^b^	*p* = **0.038^a^**
Age	*p* = 0.089^a^	*p* = 0.495^b^	*p* = 0.864^a^
Residency	*p* = 0.135^a^	*p* > 0.999^b^	*p* = 0.862^a^
Education level	*p* = 0.856^b^	*p* = 0.515^b^	*p* = 0.439^a^
Employment status	*p* = 0.407^b^	*p* > 0.999^b^	*p* = 0.059^b^
Marital status	*p* = 0.666^a^	*p* > 0.999^b^	*p* = 0.860^a^
Self-perceived economic status	*p* = **0.001^a^**	*p* = 0.441^b^	*p* = **0.025^a^**
Religiousness	*p* < **0.001^b^**	*p* = 0.234^b^	*p* = 0.156^a^
**Pre-RTA health status**			
Smoking	*p* = 0.669^a^	*p* = 0.729^b^	*p* = 0.597^a^
Alcohol use	*p* = 0.833^a^	*p* = 0.743^b^	*p* = 0.169^a^
Psychoactive substance use	*p* = 0.174^b^	*p* = 0.058^b^	*p* = 0.323^a^
Previous RTA experience	*p* = 0.202^a^	*p* = 0.308^b^	*p* = 0.226^a^
Previous traumatic experience	*p* = 0.057^a^	*p* = 0.174^b^	*p* = 0.395^a^
Previous PTSD	*p* = 0.588^b^	*p* > 0.999^b^	*p* = 0.658^b^
Previous chronic illness	*p* = 0.054^a^	*p* = **0.035^b^**	*p* = 0.169^a^
Previous psychiatric illness	*p* = 0.999^b^	*p* = **0.011^b^**	*p* = **0.008^a^**
Previous permanent pain	*p* = 0.299^b^	*p* < **0.001^b^**	*p* > 0.999^b^
Medication use	*p* = **0.001^a^**	*p* = 0.169^b^	*p* = **0.002^a^**
Type of medication used	*p* = **0.002^b^**	*p* = **0.010^b^**	*p* < **0.001^b^**
**Injury-related**			
Injury affliction	*p* = 0.741^b^	*p* > 0.999^b^	*p* = **0.013^a^**
Injury severity	*p* < **0.001^b^**	*p* = 0.524^b^	*p* = **0.004^a^**
Self-perceived threat to life	*p* = 0.087^a^	*p* = **0.040^b^**	*p* = **0.002^a^**
Pain after RTA	*p* = 0.200^a^	*p* = 0.208^b^	*p* = **0.006^a^**
Hospitalization	*p* = **0.006^a^**	*p* = 0.719^b^	*p* = 0.358^a^
Surgical treatment	*p* = **0.001^b^**	*p* > 0.999^b^	*p* = 0.564^b^
Unconsciousness in the RTA	*p* = **0.020^b^**	*p* > 0.999^b^	*p* = 0.254^b^
Post-RTA amnesia	*p* = **0.015^b^**	*p* = 0.356^b^	*p* = 0.057^a^
Hospitalization duration	*p* = **0.009^b^**	*p* = 0.671^b^	*p* = **0.011^b^**
**RTA-related**			
Fault in the RTA	*p* = 0.278^b^	*p* = 0.227^b^	*p* = **0.044^b^**
Deaths in the RTA	*p* = 0.319^b^	*p* > 0.999^b^	*p* = 0.543^b^
Compensation claim	*p* = 0.284^a^	*p* = 0.171^b^	*p* < **0.001^a^**
Road user type	*p* = 0.085^b^	*p* = 0.094^b^	*p* = 0.177^b^

^a^ Chi-square test; ^b^ Fisher’s exact test. Bold *p* values are statistically significant.

## References

[1] World Health Organization (2018). Global Status Report on Road Safety 2018.

[2] GBD 2013 DALYs and HALE Collaborators (2015). Global, regional and national disability-adjusted life years (DALYs) for 306 diseases and injuries and healthy life expectancy (HALE) for 188 countries, 1990–2013: Quantifying the epidemiological transition. Lancet.

[3] World Health Organization (2015). Global Status Report on Road Safety 2015.

[4] Croatian Bureau of Statistics (2019). Registered Road Vehicles and Road Traffic Accidents in 2018.

[5] Republic of Croatia, Ministry of Internal Affairs (2018). Bulletin on Road Traffic Safety in 2017.

[6] Hours M., Chossegros L., Charnay P., Tardy H., Nhac-Vu H.T., Boisson D., Luaute J., Laumon B. (2013). Outcomes one year after a road accident: Results from the ESPARR cohort. Accid. Anal. Prev..

[7] Chossegros L., Hours M., Charnay P., Bernard M., Fort E., Boisson D., Sancho P.O., Yao S.N., Laumon B. (2011). Predictive factors of chronic post-traumatic stress disorder 6 months after a road traffic accident. Accident Anal. Prev..

[8] Copanitsanou P., Drakoutos E., Kechagias V. (2018). Posttraumatic stress, depressive emotions, and satisfaction with life after a road traffic accident. Otrhop. Nurs..

[9] Kenardy J., Edmed S.L., Shourie S., Warren J., Crothers A., Brown E.A., Cameron C.M., Heron-Delaney M. (2018). Changing patterns in the prevalence of posttraumatic stress disorder, major depressive episode and generalized anxiety disorder over 24 months following a road traffic crash: Results from the UQ SuPPORT study. J. Affect. Disord..

[10] Ning L., Guan S., Liu J. (2017). Impact of personality and social support on posttraumatic stress disorder after traffic accidents. Medicine.

[11] Khodadadi-Hassankiadeh N., Nayeri N.D., Shahsavari H., Yousefzadeh-Chabok S., Haghani H. (2017). Predictors of post-traumatic stress disorder among victims of serious motor vehicle accidents. Int. J. Community Based Nurs. Midwifery.

[12] Hruska B., Irish L.A., Pacella M.L., Sledjeski E.M., Delahanty D.L. (2014). PTSD symptom severity and psychiatric comorbidity in recent motor vehicle accident victims: A latent class analysis. J. Anxiety Disord..

[13] Guest R., Tran Y., Gopinath B., Cameron I.D., Craig A. (2018). Prevalence and psychometric screening for detection of major depressive disorder and post-traumatic stress disorder in adults injured in a motor vehicle crash who are engaged in compensation. BMC Psychol..

[14] Yohannes K., Gebeyehu A., Adera T., Ayano G., Fekadu W. (2018). Prevalence and correlates of post-traumatic stress disorder among survivors of road traffic accidents in Ethiopia. Int. J. Ment. Health Syst..

[15] Asuquo J.E., Edet B.E., Abang I.E., Essien E.A., Osakwe O.G., Aigbomain E.J., Chigbundu K.C. (2017). Depression and posttraumatic stress disorder among road traffic accident victims managed in a Tertiary hospital in Southern Nigera. Niger. J. Clin. Pract..

[16] Dickov A., Martinović-Mitrović S., Vučković N., Siladji-Mladenović D., Mitrović D., Jovičević M., Mišić-Pavkov G. (2009). Psychiatric consequences of stress after a vehicle accident. Psychiat. Danub..

[17] Lin W., Gong L., Xia M., Dai W. (2018). Prevalence of posttraumatic stress disorder among road traffic accident survivors. A PRISMA compliant meta-analysis. Medicine.

[18] Craig A., Tran Y., Guest R., Gopinath B., Jagnoor J., Bryant R., Collie A., Tate R., Kenardy J., Middleton J.W. (2016). Psychological impact of injuries sustained in motor vehicle crashes: Systematic review and meta-analysis. BMJ Open.

[19] Craig A., Elbers N.A., Jagnoor J., Gopinath B., Kifley A., Dinh M., Pozzato I., Ivers R.Q., Nicholas M., Cameron I.D. (2017). The psychological impact of traffic injuries sustained in a road crash by bicyclists: A prospective study. Traffic Inj. Prev..

[20] Papadakaki M., Ferraro O.E., Orsi C., Otte D., Tzamalouka G., von-der-Geest M., Lajunen T., Ozkan T., Morandi A., Sarris M. (2017). Psychological distress and physical disability in patients sustaining severe injuries in road traffic crashes: Results from a one-year cohort study from three European countries. Injury.

[21] Heron-Delaney M., Kenardy J., Charlton E., Matsuoka Y. (2013). A systematic review of predictors of posttraumatic stress disorder (PTSD) for adult road traffic crash survivors. Injury.

[22] Tournier C., Charnay P., Tardy H., Chossegros L., Carnis L., Hours M. (2014). A few seconds to have an accident, a long time to recover: Consequences for road accident victims from the ESPARR cohort 2 years after the accident. Accid. Anal. Prev..

[23] Kupchik M., Strous R.D., Erez R., Gonen N., Weizman A., Spivak B. (2007). Demographic and clinical characteristics of motor vehicle accident victims in the community general health outpatient clinic: A comparison of PTSD and non-PTSD subjects. Depress. Anxiety.

[24] Stein D.J., Karam E.G., Shahly V., Hill E.D., King A., Petukhova M., Atwoli L., Bromet E.J., Florescu S., Haro J.M. (2016). Post-traumatic stress disorder associated with life-threatening motor vehicle collisions in the WHO World Mental Health Surveys. BMC Psychiatry.

[25] Littleton S.M., Hughes D.C., Poustie S.J., Robinson B.J., Neeman T., Smith P.N., Cameron I.D. (2012). The influence of fault on health in the immediate post-crash period following road traffic crashes. Injury.

[26] Ehring T., Ehlers A., Gluksman E. (2008). Do cognitive models help in predicting the severity of posttraumatic stress disorder, phobia, and depression after motor vehicle accidents? A prospective longitudinal study. J. Consult. Clin. Psychol..

[27] Jagnoor J., Blyth F., Gabbe B., Derrett S., Boufous S., Dinh M., Day R., Button G., Gillet M., Joseph T. (2014). Factors influencing social and health outcomes after motor vehicle crash injury: An inception cohort study protocol. BMC Public Health.

[28] Civil I.D., Schwab C.W. (1988). The Abbreviated Injury Scale, 1985 Revision: A Condensed Chart for Clinical Use. J. Traum..

[29] Stevenson M., Segui-Gomez M., Lescohier I., Di Scala C., McDonald-Smith G. (2001). An overview of the injury severity score and the new injury severity score. Inj. Prev..

[30] Blanchard E.B., Jones-Alexander J., Buckley T.C., Forneris C.A. (1996). Psychometric properties of the PTSD Checklist (PCL). Behav. Res. Ther..

[31] National Center for PTSD Using the PTSD checklist (PCL). https://sph.umd.edu/sites/default/files/files/PTSDChecklistScoring.pdf.

[32] Beck A.T., Epstein N., Brown G., Steer R.A. (1988). An inventory for measuring clinical anxiety: Psychometric properties. J. Consult. Clin. Psych..

[33] Beck A.T., Steer R.A., Garbin M.G. (1988). Psychometric properties of the Beck Depression Inventory: Twenty-five years of evaluation. Clin. Psychol. Rev..

[34] Elbers N.A., Akkermans A.J., Lockwood K., Craig A., Cameron I.D. (2015). Factors that challenge health for people involved in the compensation process following a motor vehicle crash: A longitudinal study. BMC Public Health.

[35] European Commission Mobility and Transport Road Safety. https://ec.europa.eu/transport/road_safety/specialist/knowledge/safetyratings/changing_design_upgrading_standards_and_reducing_casualties/in_car_safety_en.

[36] Croatian Centre for vehicles Technical examination. Statistics. https://www.cvh.hr/media/3012/s01__pregled_starosti_vozila_prema_vrstama_vozila_2018.pdf.

[37] Hasselberg M., Kirsebom M., Backstorm J., Berg H.Y., Rissanen R. (2019). I did NOT feel like this at all before the accident: Do men and women report different health and life consequences of a road traffic injury?. Inj. Prev..

[38] Giummarra M., Black O., Smith P., Collie A., Hassani-Mahmooei B., Arnold C.A., Gong J., Gabbe B.J. (2018). A population-based study of treated mental health and persistent pain conditions after transport injury. Injury.

[39] Littleton S.M., Cameron I.D., Poustie S.J., Hughes D.C., Robinson B.J., Neeman T., Smith P.N. (2011). The association of compensation on longer term health status for people with musculosceletal injuries following road traffic crashes: Emergency department inception cohort study. Injury.

[40] Ravn S.L., Hartvigsen J., Hansen M., Sterling M., Andresen T.E. (2018). Do post-traumatic pain and post-traumatic stress symptomatology mutually maintain each other? A systematic review of cross-lagged studies. Pain.

[41] Jagnoor J., De Wolf A., Nicholas M., Maher C.G., Casey P., Blyth F., Harris I.A., Cameron I.D. (2015). Restriction in functioning and quality of life is common in people 2 months after compensable motor vehicle crashes: Prospective cohort study. Inj. Epidemiol..

[42] Murgatroyd D.F., Harris I.A., Tran Y., Cameron I.D. (2016). The association between seeking financial compensation and injury recovery following motor vehicle related orthopaedic trauma. BMC Musculoskel. Dis..

[43] Undavalli C., Das P., Dutt T., Bhoi S., Kashyap R. (2014). PTSD in post-traffic accident patients requiring hospitalization in Indian subcontinet: A review on magnitude of the problem and management guidelines. J. Emerg. Trauma Shock.

